# Altered Expression of Genes Implicated in Xylan Biosynthesis Affects Penetration Resistance against Powdery Mildew

**DOI:** 10.3389/fpls.2017.00445

**Published:** 2017-03-31

**Authors:** Jamil Chowdhury, Stefanie Lück, Jeyaraman Rajaraman, Dimitar Douchkov, Neil J. Shirley, Julian G. Schwerdt, Patrick Schweizer, Geoffrey B. Fincher, Rachel A. Burton, Alan Little

**Affiliations:** ^1^ARC Centre of Excellence in Plant Cell Walls, School of Agriculture, Food and Wine, University of AdelaideGlen Osmond, SA, Australia; ^2^Pathogen-Stress Genomics, Leibniz Institute of Plant Genetics and Crop Plant Research (IPK)Stadt Seeland, Germany

**Keywords:** cell wall, xylan, papillae, glycosyltransferase, fungi, powdery mildew, *Blumeria graminis*, penetration

## Abstract

Heteroxylan has recently been identified as an important component of papillae, which are formed during powdery mildew infection of barley leaves. Deposition of heteroxylan near the sites of attempted fungal penetration in the epidermal cell wall is believed to enhance the physical resistance to the fungal penetration peg and hence to improve pre-invasion resistance. Several glycosyltransferase (GT) families are implicated in the assembly of heteroxylan in the plant cell wall, and are likely to work together in a multi-enzyme complex. Members of key GT families reported to be involved in heteroxylan biosynthesis are up-regulated in the epidermal layer of barley leaves during powdery mildew infection. Modulation of their expression leads to altered susceptibility levels, suggesting that these genes are important for penetration resistance. The highest level of resistance was achieved when a GT43 gene was co-expressed with a GT47 candidate gene, both of which have been predicted to be involved in xylan backbone biosynthesis. Altering the expression level of several candidate heteroxylan synthesis genes can significantly alter disease susceptibility. This is predicted to occur through changes in the amount and structure of heteroxylan in barley papillae.

## Introduction

The cell wall is a dynamic structural barrier that can determine the outcome of the interactions between plants and pathogens. At the sites of interaction, plants actively reinforce the cell wall though development of cell wall appositions, called papillae (Zeyen et al., 2002). Papillae formation appears to be a consequence of pathogen-associated molecular pattern (PAMP)-triggered immunity (PTI) and/or damage-associated molecular pattern (DAMP)-triggered immunity (DTI), that is observed during many plant-pathogen interactions (Boller and Felix, 2009; Malinovsky et al., 2014). Although, the role of papillae is not fully understood, it is believed that they provide a physical and chemical barrier that can completely prevent pathogen entry or at least delay pathogen penetration into the cells so that other defense strategies can be activated (Stone and Clarke, 1992; Huckelhoven, 2005). Without successful penetration the pathogen is unable to develop a feeding structure called the haustorium which is required to absorb water and nutrients from the plant (Bélanger et al., 2002). The composition of papillae may vary among different species, but commonly found papillae components are cell wall polysaccharides, cell wall proteins, reactive oxygen species, phenolics and anti-microbial compounds (Zeyen et al., 2002; Hückelhoven, 2014). Among the cell wall polysaccharides, callose has commonly been assumed to be the major polysaccharide in the papillae that form during plant/pathogen interactions, but it has recently been shown that arabinoxylan and cellulose are also major constituents of papillae that form during infection of barley by the powdery mildew pathogen *Blumeria graminis* f. sp. *hordei* (*Bgh*) (Chowdhury et al., 2014). Earlier and heavier deposition of these polysaccharides are proposed to aid in arresting the progress of the fungal penetration peg through effective papillae formation (Chowdhury et al., 2014). A glucan synthase gene (*HvGsl6*) and a cellulose synthase-like D2 gene (*HvCslD2*) have been shown to contribute toward the biosynthesis of callose and cellulose in barley papillae, respectively, and down regulation of these genes results in increased penetration susceptibility to *Bgh* (Chowdhury et al., 2016; Douchkov et al., 2016). Together these results suggest that polysaccharide deposition in papillae is essential for the mechanism of penetration resistance in barley against powdery mildew infection.

Arabinoxylan was detected in barley papillae using the LM11 monoclonal antibody, which binds to an epitope of low substituted xylan, whereas no significant labeling was observed using the LM10 antibody, which binds unsubstituted xylan (Chowdhury et al., 2014). The arabinoxylan epitopes were distributed throughout the entire papillae, in which two zones could be readily recognized. An inner core consisted of arabinoxylan, callose and phenolics, while an outer layer, or coat, contained arabinoxylan and cellulose. As the LM11 antibody binds to wheat arabinoxylan with a low degree of arabinose substitutions (McCartney et al., 2005), we can assume that some degree of substitution of the backbone xylan with arabinosyl residues is likely to be present in barley papillae. However, we cannot be sure what other substitutions may occur along the xylan backbone and will therefore refer to the polysaccharide as heteroxylan.

Cell wall reinforcement in response to biotrophic and necrotrophic pathogen attack can be mediated through oxidative cross-linking between polysaccharides, structural proteins and phenolic compounds (Passardi et al., 2004; Deepak et al., 2010). The presence of heteroxylan in papillae provides an opportunity for increased cell wall polymer cross-linking through associated phenolic compounds, such as ferulic acid. More highly cross-linked wall polysaccharides would presumably toughen the wall and increase resistance against fungal penetration, whether that penetration is mediated through physical pressure or by enzymatic hydrolysis. In monocotyledonous plants, ferulic acid can be ester-linked to arabinofuranosyl substituents of the xylan backbone. The ferulic acid may undergo oxidative dimerization to covalently link adjacent feruloylated arabinoxylan chains or, alternatively, the heteroxylan chains might be cross-linked with lignin (Burr and Fry, 2009; Marcia, 2009). There is evidence that arabinoxylan with low degrees of substitution can interact with cellulose microfibrils and other polymers via extensive intermolecular hydrogen bonding (Busse-Wicher et al., 2014) and this is also likely to influence the strength and elasticity of walls, and contribute to the resistance of the walls to enzymatic degradation. It can be argued that heteroxylan, as a major component of papillae, helps to provide mechanical strength against the fungal penetration process. Thus, genetic manipulation of heteroxylan content or structure may impact upon the plant's susceptibility to infection.

**Figure 1:**
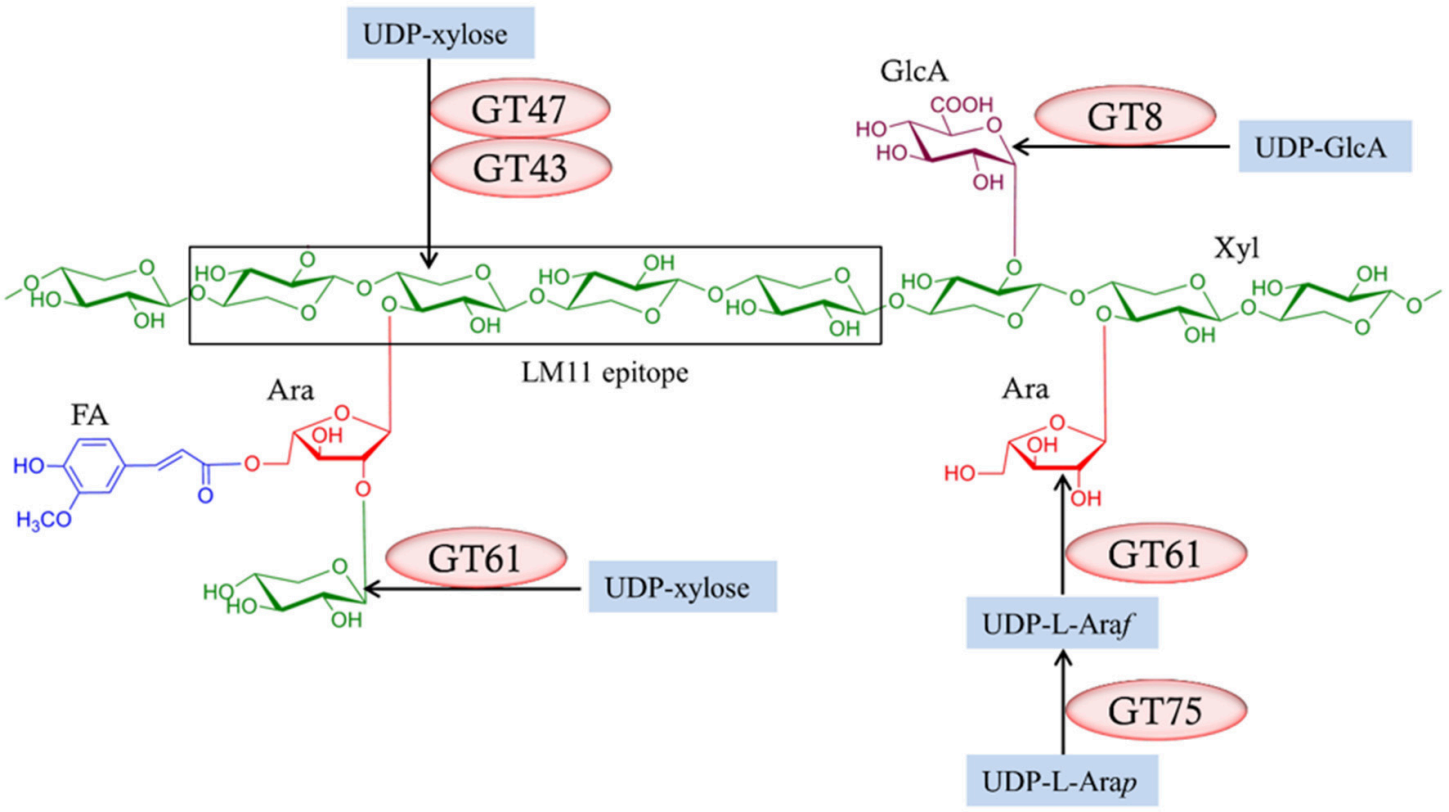
**Predicted roles of glycosyltransferase (GT) gene families in arabinoxylan biosynthesis**. The LM 11 antibody recognizes an arabinoxylan backbone carrying a low degree of arabinose substitutions. Xyl, Xylose; Ara, Arabinose; GlcA, Glucuronic acid; FA, Ferulic acid. UDP-L-Ara*p*, UDP-L-arabinopyranose; UDP-L-Ara*f*, UDP-L-arabinofuranose.

The complete process of heteroxylan biosynthesis in the plant cell wall is not fully understood. However, there is evidence to suggest that the assembly of the xylan backbone and its substituents involves at least six families of glycosyltransferases (GTs) found in the Carbohydrate-Active EnZymes (CAZy) database (Cantarel et al., 2009), namely GT8, GT31, GT43, GT47, GT61, and GT75 (Rennie and Scheller, 2014). The predicted role for each of these GT families in assembling heteroxylan in grasses is presented in Figure 1. Two members of the GT43 family, known as *Irregular Xylem* 9 (*IRX9*) and *IRX14*, and a member of the GT47 family, *IRX10*, have putative xylosyltransferase activities required for synthesizing the xylan backbone (Brown et al., 2007; Lee et al., 2007; Wu et al., 2009; Mortimer et al., 2015). Two members of the GT8 family, termed *Glucuronic Acid Substitution of Xylan* (*GUX*) *1* and *GUX2*, introduce glucuronyl (GlcA) substituents onto the xylan backbone (Mortimer et al., 2010; Rennie et al., 2012). Members of the GT75 family, which encode UDP-arabinose mutases (UAM) or Reversibly Glycosylated Protein (RGP), are responsible for converting UDP-arabinopyranose to UDP-arabinofuranose, which is the substrate for xylan arabinosyltransferases (Rautengarten et al., 2011; Hsieh et al., 2016). Some members of the GT61 family have xylan arabinosyltransferase activity that adds arabinosyl residues onto the xylan backbone (Anders et al., 2012), while another member of the GT61 family is capable of mediating xylosyl substitution of arabinosyl residues on the xylan backbone (Chiniquy et al., 2012). The GT31 family was also included in our study due to its reported association with heteroxylan synthesis, although there is no direct evidence that these enzymes are directly involved. Several GT31 genes encode galactosyltransferases that transfer galactosyl residues from UDP-galactose to arabinogalactan-proteins (AGPs) (Nguema-Ona et al., 2015). AGPs may be cross-linked with heteroxylan (Tan et al., 2013) and may also play a role in pathogen defense responses (Nguema-Ona et al., 2013).

In the current study, we have investigated the potential roles in penetration resistance against *Bgh* of genes that have been implicated in heteroxylan biosynthesis. Up- or down-regulation of transcript levels of selected candidate genes during infection indicated that the susceptibility against *Bgh* penetration is significantly altered in barley epidermal cells. The identification of these genes might provide new opportunities for the generation of novel crop lines with greater disease resistance.

## Materials and methods

### Plant and fungal materials

Australian and Swiss isolates of *B. graminis* f. sp. *hordei* (*Bgh*) were provided by Prof. Richard Oliver, Centre for Crop Disease Management, Curtin University, Western Australia and by Prof. Robert Dudler, University of Zürich, Switzerland, respectively. The Swiss field isolate FAL 92315 of *B. graminis* f. sp. *tritici* (*Bgt*) was provided by Prof. Robert Dudler, University of Zürich, Switzerland. The barley and wheat isolates were maintained on 7-day-old seedlings of susceptible barley cultivars “Baudin,” “Golden Promise,” or on wheat cultivar Kanzler as described previously (Chowdhury et al., 2014; Douchkov et al., 2014).

### Phylogenetic relationship analysis

Clustal Omega (Sievers et al., 2011) was used to align amino acid sequences with the following guide hidden markov models: PTHR11214 (GT31), PTHR10896 (GT43), PTHR11062 (GT47), PTHR20961 (GT61), and Glyco_transf_8.hmm (GT8). Alignments were trimmed using BMGE (Criscuolo and Gribaldo, 2010) to carry forward only those sites whose variation can be categorized as biologically meaningful. Final gamma based likelihood values from RAxML8 (Stamatakis, 2014) were assessed using PROTGTRCAT, PROGTRGAMMAX, PROGTRCATX, PROTLGGAMMA, PROTLGGAMMAX, PROTLGCAT, PROTLGCATX substitution models. The PROTGTRGAMMAX amino acid substitution model produced the highest likelihood for all data and out-performed best fit empirical models. All RAxML analyses began with a 1,000 rapid bootstrap analysis and 200 best ML tree search. The tree with the highest likelihood was then used as the starting tree for 1,000 rapid hill-climb ML tree searches and 1,000 randomized tree searches. This procedure was repeated three times for each dataset and the trees with the highest final gamma-based likelihood were chosen.

### Microarray transcript profiling

The transcript profiles for the genes of interest in this study were extracted from microarray data that is available at ArrayExpress (Accession E-MTAB-2916; Douchkov et al., 2014). RNA was isolated from the abaxial leaf epidermal peel of 7-day-old barley plants cv. Vada inoculated with *B. graminis* f. sp. *hordei* (*Bgh*) or *B. graminis* f. sp. *tritici* (*Bgt*) at 6–74 h after inoculation as described previously (Zellerhoff et al., 2010). Total quality-controlled RNA was hybridized to a 44 K Agilent oligonucleotide array chip (Chen et al., 2011). Raw Details of the data normalization procedure have been described previously (Douchkov et al., 2014). In order to identify differentially-regulated transcripts, two types of statistical analysis were carried out using GeneSpring GX (v11.5.1) software (Agilent technologies Inc). First, a paired *t*-test was carried comparing the inoculated vs. non-inoculated samples across all time-points. Second, to identify additional transcripts that were transiently regulated at a particular time point, an unpaired *t*-test and a one way analysis of variance (ANOVA) was performed at each individual time point respectively, comparing inoculated vs. non-inoculated sample. In general, transcripts were assumed to be significantly regulated if the *p*-value corrected for false-discovery rate [Benjamini–Hochberg multiple testing correction for *t*-tests and Tukey honestly significant difference *post-hoc* test (HSD-test) for ANOVA] was smaller than or equal to 0.05 (*p* ≤ 0.05) and if regulation factors (fold change; FC) between inoculated and corresponding non-inoculated samples harvested in parallel exceeded 2.0 (FC ≤ 2). Transcripts that passed the first or second set of statistical criteria were pooled to form a non-redundant list.

### Biolistic DNA delivery for transiently induced gene silencing and transient over-expression

To test the impacts of over-expression or silencing of the genes of interest on the penetration success of *Bgh* in barley leaf epidermal cells, DNA-coated gold particles carrying a reporter gene as well as a test gene construct were co-bombarded using a biolistic DNA delivery system as outlined in Schweizer et al. (1999b), with modifications (Chowdhury et al., 2016). Quantification of the pathogen susceptibility index in several previous studies in relation to a control gene transformation indicates the impact of the gene of interest on the outcome of the plant pathogen interaction (Nielsen et al., 1999; Schweizer et al., 1999a; Panstruga, 2004; Douchkov et al., 2014). For transiently induced gene silencing (TIGS) experiments, ~500 bp of the candidate gene sequences were PCR-amplified using the primers listed in Table S2, and ligated into pIPKTA38 entry clones. The cloned fragments were recombined into the RNA interference (RNAi) destination vector pIPKTA30N as inverted repeats, using the Gateway LR clonase reaction (Invitrogen) according to the procedure described in Douchkov et al. (2005). Possible off-target effects from use of the transiently induced gene silencing constructs were predicted using si-Fi software (labtools.ipk-gatersleben.de). Several previously tested genes (*mlo, HvSNAP34*, and *HvCslD2*) were used as positive control genes for transient gene expression assays because they are known to have a significant impact on pathogen susceptibility of transformed cells upon their knockdown regulation (Douchkov et al., 2014). A PDS-100/He microprojectile bombardment system was used to co-bombard 7-day-old detached barley leaves of cv. Golden Promise with the candidate gene dsRNAi constructs together with a vector containing the reporter gene β-glucuronidase (GUS; Douchkov et al., 2005). Seventy two hours post bombardment the leaf segments were inoculated with powdery mildew conidia of Swiss *Bgh* isolate CH4.8 at a density of ~200 conidia/mm^2^. Forty eight hours post inoculation, the relative susceptibility index (RSI) was calculated by dividing the number of cells containing haustoria by the total number of transformed cells expressing the GUS reporter gene, and the data were normalized against the empty vector control that had a susceptibility index ranging from 5 to 14%.

In transient over-expression experiments, the longest coding sequence of the candidate genes was obtained from a publicly available database (plants.ensembl.org). Candidate genes of interest were PCR-amplified using the Phusion polymerase (New England Biolab® Inc., Ipswich, USA) with primers flanking the predicted coding region (Table S3). The amplified sequences were ligated into the pCR8 entry vector (Invitrogen) and recombined into a Gateway enabled destination vector, pEAQ-*HT*-DEST1 (Sainsbury et al., 2009) where genes of interest are under the control of a CaMV 35S promoter. The same conditions were used for microprojectile bombardment in silencing and over-expression experiments, except that a Green Fluorescent Protein (GFP) expressing construct was used instead of the GUS reporter construct. Seventy two hours post bombardment the leaf segments were inoculated with powdery mildew conidia of the Australian *Bgh* isolate at a density of ~200 conidia/mm^2^. Forty eight hours post inoculation, the relative susceptibility index (RSI, compared to the empty-vector control that was set to 100%) was calculated, following the method described previously (Douchkov et al., 2014). Haustoria present in GFP-transformed cells were observed in live tissue under a GFP filter (excitation 485/20 nm, emission 530/25 nm) of a fluorescence microscope (Axio Imager M2; Carl Zeiss, Oberkochen, Germany) pre-aligned with a mercury system.

## Results

### Transcript profiling of heteroxylan biosynthetic gene family members during *Bgh* infection

**Figure 2:**
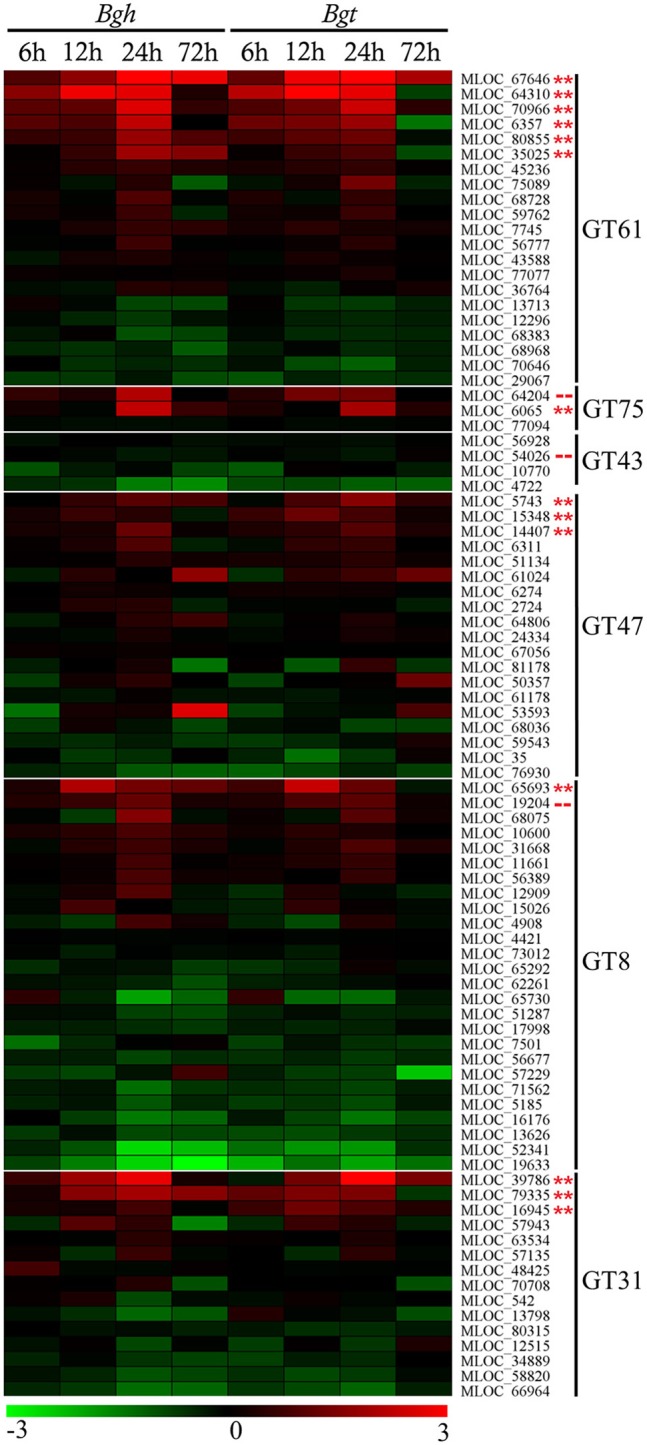
**Microarray transcript profiling of the glycosyltransferase gene families in barley leaves infected with adapted ***Blumeria graminis*** f. sp. ***hordei*** (***Bgh***) and non-adapted ***Blumeria graminis*** f. sp. ***tritici*** (***Bgt***) fungi at different time points**. The scale of this heatmap is given as standardized fold-changes (log2) of infected tissue relative to the uninfected control with a range from −3 (green) to 3 (red). Individual constructs with a double asterisk (^**^) displayed a statistically significant (*p* < 0.05, paired *t*-test, unpaired *t*-test and ANOVA) increase in transcript levels, while constructs with a dashed line (- -) were selected for further analysis on the basis of predicted function and basal expression levels. A list of the genes with their corresponding microarray probeset ID is available in Table S1.

The transcript expression profiles of candidate GT gene families induced in the barley epidermal layer during infection by the adapted *B. graminis* f. sp. *hordei* (*Bgh*, 25% susceptible epidermal cells) and the non-adapted *B. graminis* f. sp. *tritici* (*Bgt*, 0% susceptible epidermal cells) were quantified using data extracted from a previous microarray analysis (ArrayExpress Accession E-MTAB-2916; Douchkov et al., 2014; Rajaraman, 2016). As shown in Figure 2 a relatively small number of each GT family were significantly up-regulated; most members showed no significant changes while a few members were down-regulated. The development of papillae under the first and second appressorial lobes was completed within 24 h after inoculation, and the gene candidates were therefore selected on the basis of their up-regulation patterns within this time frame. With few exceptions, the transcript patterns of the up-regulated gene family members were comparable during *Bgh* and *Bgt* infection. The members of each family showing the highest increase were selected for further analysis. Among the selected GT families, the GT43 family was the only family that did not show increases in expression during powdery mildew infection. Due to the predicted involvement of the GT43 family in xylan synthesis, one candidate gene from the GT43 family (MLOC_54026) was selected for further analysis. Given that the transcripts of this gene were highly abundant in both infected and uninfected epidermal tissue it was considered that it might still play a role in the synthesis of the papillae.

To assign putative functions to the selected candidate genes, phylogenetic analyses of each GT family were performed, using protein sequences from the barley, rice and *Arabidopsis* genomes (Figures S1–S6). The annotated functions of the selected barley candidates are summarized in Table 1.

**Table 1:** **Selected candidate genes and their putative function in the synthesis of arabinoxylan**.

**MLOC ID**	**CAZy family**	***Arabidopsis* homolog**	**Putative function**	Chromosome number^*^	Chromosome position^*^ (cM)	**Host epi. transcript regulation (Log2 fold change 24hai)**	**Non-host epi. transcript regulation (Log2 fold change 24hai)**	**References**
MLOC_54026	GT43	AT1G27600 (IRX9-L)	β-1,4 Xylosyl transferase	1H	116.501	NS (0.03)	NS (−0.17)	Lee et al., 2010; Mortimer et al., 2015
MLOC_5743	GT47	AT3G45400	Exostosin family protein with unknown function	4H	51.404	UP (1.04)	UP (1.60)	Andersson-Gunnerås et al., 2006
MLOC_15348	GT47	AT2G20370 (MUR3)	Xyloglucan galactosyl transferase, actin organization	4H	91.713	UP (0.46)	UP (0.80)	Chevalier et al., 2010; Li et al., 2013
MLOC_14407	GT47	AT4G16745	Exostosin family protein with unknown function	3H	6.149	UP (1.20)	UP (1.01)	Wang et al., 2008
MLOC_65693	GT8	AT2G35710	UDP-glucuronyl transferase	1H	47.827	UP (1.37)	UP (1.19)	Rennie et al., 2012
MLOC_19204	GT8	AT5G18480	UDP-glucuronyl transferase	2H	71.956	UP (1.19)	UP (1.08)	Rennie et al., 2012, 2014
MLOC_67646	GT61	Not found	Unknown function	3H	141.918	UP (5.73)	UP (5.32)	–
MLOC_64310	GT61	AT3G18170AT3G18180	Arabinofuranosyl transferase (homolog of TaXAT1)	6H	72.238	UP (2.70)	UP (2.73)	Anders et al., 2012
MLOC_70966	GT61	Not found	Unknown function	7H	67.917	UP (2.62)	UP (2.41)	–
MLOC_6357	GT61	AT3G18170AT3G18180	Arabinofuranosyl transferase	7H	69.263	UP (2.25)	UP (1.80)	Anders et al., 2012
MLOC_80855	GT61	AT3G18170AT3G18180	Arabinofuranosyl transferase	6H	49.787	UP (1.79)	UP (1.27)	Anders et al., 2012
MLOC_35025	GT61	AT3G10320	Xylosyl transferase, decorates xylan with xylose side chains	1H	17.288	UP (1.86)	UP (0.91)	Voiniciuc et al., 2015
MLOC_64204	GT75	Not found	UDP-Arabinose Mutase (*HvUAM3*)			UP (2.09)	UP (1.34)	Hsieh et al., 2016
MLOC_6065	GT75	Not found	UDP-Arabinose Mutase (*HvUAM1*)	4H	51.274	UP (2.26)	UP (1.95)	Hsieh et al., 2016
MLOC_39786	GT31	AT5G57500	Galactosyl transferase	5H	55.625	UP (2.75)	UP (3.42)	Qu et al., 2008
MLOC_79335	GT31	AT1G27120	Hydroxyproline-O-galactosyl transferase	5H	143.403	UP (1.94)	UP (1.48)	Qu et al., 2008; Basu et al., 2015
MLOC_16945	GT31	AT1G27120	Hydroxyproline-O-galactosyl transferase	1H	18.272	UP (0.78)	UP (0.91)	Qu et al., 2008; Basu et al., 2015

* *The physical location of the candidate genes derived from different mapping populations: Morex × Barke POPSEQ 2013, Oregon Wolfe POPSEQ 2013 extracted from morexGenes—Barley RNA-seq Database of The James Hutton Institute, hai, hours after inoculation (IBGSC, 2012)*.

### Altered pathogen susceptibility following transiently induced gene silencing of candidate genes

**Figure 3:**
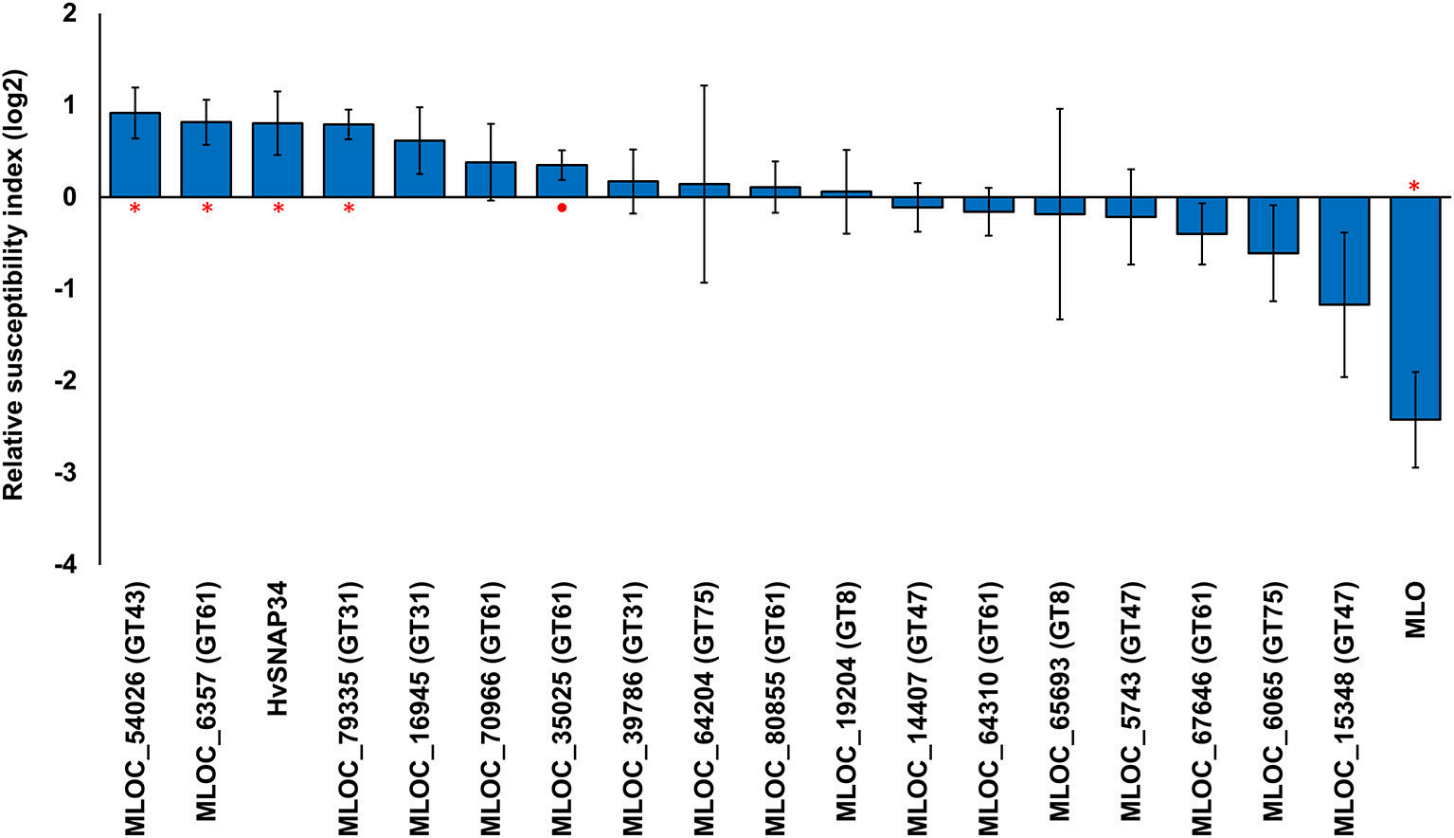
**Transient induced gene silencing of candidate genes and the changes in susceptibility to fungal penetration**. The relative susceptibility index (RSI) was calculated as fold-changes (log2) normalized against the empty vector control. Individual constructs with an asterisk (^*^) displayed a statistically significant (*p* < 0.05, one sample *t*-test) difference compared to the empty vector construct, while constructs with a solid circle (•) were close to the statistical cut-off (0.05 < *p* < 0.1). Each construct was performed with a minimum of three replicates. Error bars indicate standard error.

To examine the potential roles of candidate genes for arabinoxylan biosynthesis, we conducted transiently induced gene silencing (TIGS) experiments (Douchkov et al., 2014), which allow the examination of the susceptibility of papillae in barley epidermal cells to penetration by *Bgh* when the candidate genes are down-regulated. In this experiment dsRNAi silencing constructs targeting each candidate gene were expressed in the barley leaf following biolistic bombardment. The bombarded cells were subsequently tested for their ability to prevent fungal penetration and subsequent formation of haustoria. The relative susceptibility index (RSI) was used to measure the ratio of successful fungal penetrations compared with the empty vector control. Down-regulation of three to four candidate genes led to significantly increased susceptibility, depending on significance thresholds chosen (Figure 3). The maximum shift in the susceptibility level was achieved by silencing a GT43 gene (MLOC_54026), which resulted in a RSI of 0.92 (189%) compared with the control, followed by a GT61 (MLOC_6357) at 0.82 (176%) and a GT31 gene (MLOC_79335) at 0.79 (173%; Figure 3). No significant difference was found in the RSI following silencing of the remaining candidate genes. As expected, the down-regulation of the control gene *HvSNAP34* resulted in a significant increase in susceptibility and down-regulation of *HvMlo* also resulted in a significant increase in resistance, which is consistent with previous work (Douchkov et al., 2014).

### Altered pathogen susceptibility following transient over-expression of candidate genes

**Figure 4:**
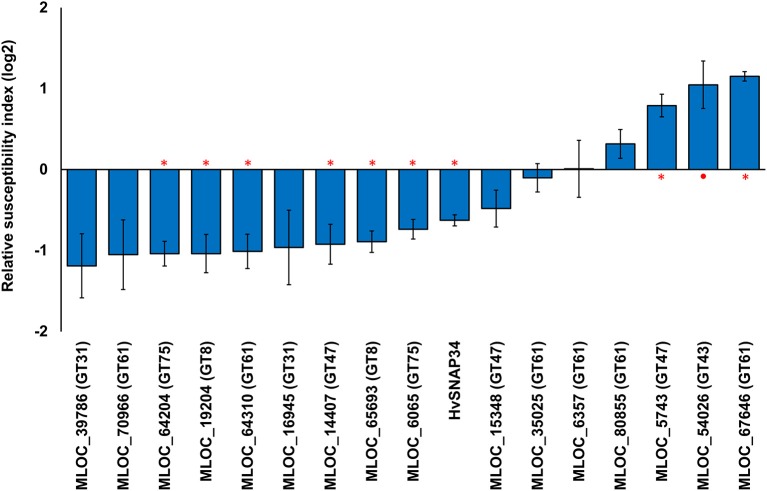
**Transient over-expression of candidate genes and the changes in susceptibility to fungal penetration**. The relative susceptibility index (RSI) was calculated as fold-changes (log2) normalized against the empty vector control. Individual constructs with an asterisk (^*^) displayed a statistically significant (*p* < 0.05, one sample *t*-test) difference compared to the empty vector construct, while constructs with a solid circle (•) were close to the statistical cut-off (0.05 < *p* < 0.1). Each construct was performed with a minimum of three replicates. Error bars indicate standard error.

Transient over-expression of candidate genes was also performed to examine the effects of putatively-increased polysaccharide levels on penetration resistance. Transient over-expression of six candidate genes led to significant decreases in the RSI compared with the empty vector (Figure 4). The maximum significant decrease in the RSI was observed by over-expressing a GT75 gene (MLOC_64204) followed by a GT8 (MLOC_19204) and a GT61 gene (MLOC_64310). The resulting RSI values were −1.04 (49%), −1.01 (50%), and 0.96 (51%) of control levels, respectively. However, over-expression of three candidate genes, MLOC_67646 (GT61), MLOC_54026 (GT43), and MLOC_5743 (GT47), resulted in increases in RSI.

**Figure 5:**
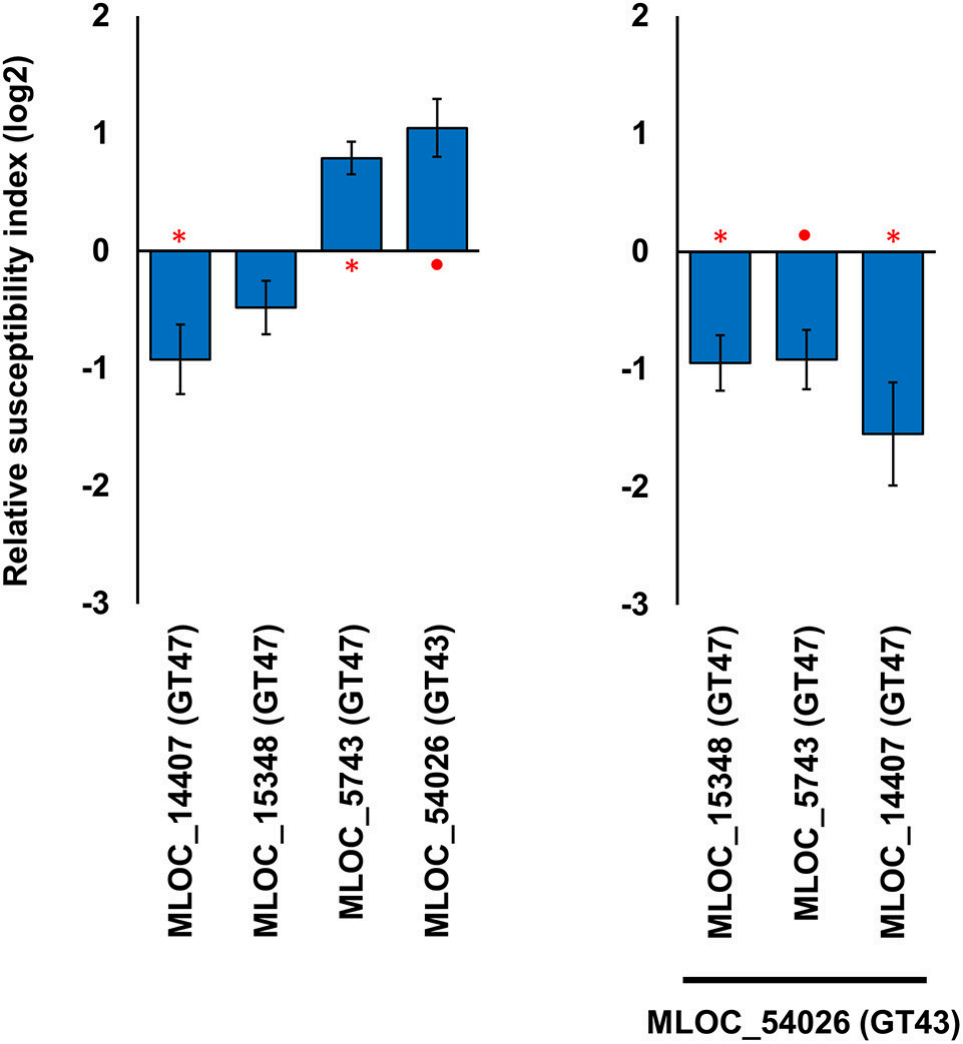
**Effects of individual and combined expression of GT43 and GT47 members on host susceptibility level**. The left hand panel shows the effects of the genes individually, and the right hand panel shows the effects of the three GT47 genes in the co-expressed with the single GT43 gene. The relative susceptibility index (RSI) was calculated as fold-changes (log2) normalized against the empty vector control. Individual constructs with an asterisk (^*^) displayed a statistically significant (*p* < 0.05, one sample *t*-test) difference compared to the empty vector construct, while constructs with a solid circle (•) were close to the statistical cut-off (0.05 < *p* < 0.1). Each construct was performed with a minimum of three replicates. Error bars indicate standard error.

Transient over-expression and TIGS did not lead to statistically significant, reciprocal phenotypes for any of the candidate genes. There may be a number of reasons why the RSI changes may vary; such as, RNAi off-target effects against related GT-family members, an inherent level of redundancy within each gene family or simply inefficient silencing by the TIGS construct. However, the data also suggest that some genes may require ancillary factors that need to be co-expressed for normal activity. Silencing of the GT43 gene (MLOC_54026) resulted in the highest level of susceptibility (RSI of 0.92, 189%), suggesting that this protein plays an integral role in papillae penetration resistance against the invading fungal pathogens. However, over-expression of this gene also resulted in the second highest observed increase in RSI (1.05, 207%). Given that any xylan synthase complex might contain both GT43 and GT47 family members, various combinations of GT43 and GT47 candidate genes were over-expressed to see if we could find any significant increase in penetration resistance. When the GT43 gene (MLOC_54026) was co-expressed with three individual members of the GT47 family, a significant increase in penetration resistance was observed in all cases. The most striking change was observed when co-expressing the two genes, GT43 (MLOC_54026) and (GT47) MLOC_5743, which individually led to increased susceptibility (RSI of 1.05, 207% and 0.79, 173%, respectively), resulted in an increase in resistance (RSI of −0.92, 53%; Figure 5). This data suggests that interactions might be occurring between the two gene products and provide indirect evidence that the two genes (MLOC_54026; GT43 and MLOC_5743; GT47) might be partners in a xylan synthase complex in epidermal cells.

## Discussion

The dynamics of heteroxylan biosynthesis represents an area of untapped potential for activating or enhancing cell wall associated plant defense mechanisms. Heteroxylan is an important component of barley papillae and is associated with penetration resistance against powdery mildew pathogens (Chowdhury et al., 2014). The precise structure of heteroxylans in papillae is not known, because it is technically difficult to isolate individual papillae for chemical analysis. However, the heteroxylans in cell walls of grasses and cereals has been shown to contain a (1,4)-β-xylan backbone and, depending upon the species and tissue type, the backbone is substituted to varying degrees with α-arabinofuranosyl (Ara*f*) residues, α-glucuronosyl residues (GlcA), and with feruloylated arabinofuranosyl residues (Carpita and Gibeaut, 1993; Stone and Fincher, 2004; Ebringerová et al., 2005; Burton and Fincher, 2012). Due to the inherent capability of feruloylated heteroxylans to form covalent cross-links via their phenolic acid residues not only with other arabinoxylan molecules, but also with other wall polymers, one might expect that this cross-linking could lead to an increase in mechanical strength of the walls and that the presence of feruloylated heteroxylans in barley papillae could be of great importance in plant resistance to fungal penetration.

To understand the role of heteroxylan in papillae-based cell wall reinforcement, we examined candidate genes selected from various glycosyltransferase (GT) families that have been implicated in arabinoxylan biosynthesis (Rennie and Scheller, 2014). A number of candidate genes from heteroxylan-associated GT families were significantly up-regulated in the barley epidermal layer during powdery mildew infection, at times corresponding to the period when heteroxylan was being synthesized in the papillae (Figure 2). Similar expression profiles were observed during infection by the non-adapted pathogen *Bgt*, suggesting that these genes are somehow involved in basal plant defense mechanisms that are not the ultimate determinants of successful penetration or resistance outcomes.

In this study, silencing and over-expression of several GT43 and GT47 candidate genes resulted in susceptibility phenotypes that might be predicted from the recent discovery that heteroxylans are present in barley papillae (Chowdhury et al., 2014). However, for all candidate genes, the predicted reciprocal resistance and susceptibility phenotypes were not obtained by up-regulation and down-regulation of their expression levels. The GT43 gene (MLOC_54026) is the barley homolog of the rice GT43E gene (LOC_Os05g48600, Figure S1), which has been demonstrated to complement the irregular xylem mutation (IRX9) in Arabidopsis (Lee et al., 2014). Mutation of the *Arabidopsis IRX9* gene (At2g37090) results in defective stem mechanical strength, vessel morphology, xylan content, GlcA side chains, xylan chain length, and xylosyltransferase activity (Lee et al., 2010; Mortimer et al., 2015). Biochemical assays for xylan xylotransferase activity demonstrated that the IRX9 protein is capable of elongation of the xylan backbone in the presence of another GT43 cofactor IRX14 (Lee et al., 2012). We have found that over-expression of this GT43 gene (MLOC_54026) in combination with members of the GT47 family leads to significant increases in resistance. The contrasting level of resistance obtained from individually over-expressing the GT43 (MLOC_54026) and GT47 (MLOC_5743) genes compared to when they are over-expressed in combination suggests that these two gene family members may somehow co-operate during the synthesis of the xylan backbone in effective papillae. This result is supported by reports that GT43 and GT47 enzymes might co-exist in a single xylan synthase complex and might need to interact for effective heteroxylan biosynthesis (Zeng et al., 2010). Although, the current data suggest that xylan represents a significant barrier to fungal penetration during the plant's defense responses, the direct impacts on disease resistance and the precise molecular mechanisms in defense-related cell walls that would explain the effects of silencing or over-expressing the GT43 and GT47 genes have yet to be defined. However, silencing of specific GT43 and GT47 gene family members can significantly improve wall digestibility by cell wall degrading enzymes in grasses and eudicots (Lee et al., 2009; Petersen et al., 2012). These results are consistent with the data presented here, which support the hypothesis that GT43 and GT47 enzymes, through their xylan biosynthesis activity, increase cell wall rigidity and hence resistance against enzymatic degradation by pathogens. It should also be pointed out that the synthesis of heteroxylans with lower degrees of substitution and/or the removal of some of the arabinosyl residues by α-arabinofuranosidases would be expected to facilitate intermolecular binding, as would an increase in ferulic acid cross-links. In both cases one might reasonably expect that the papillae would be “toughened” and would be more resistant to fungal penetration.

In addition to the GT43 and GT47 combinations, five other candidate genes produced significant resistance phenotypes when their expression levels were up-regulated. Within this group of genes were representatives of the GT8, GT61, and GT75 families. Silencing of two GT75 members (MLOC_64204 and MLOC_6065) had no significant effect on pathogen susceptibility levels, possibly due to redundancy. However, over-expression of these two genes individually led to a significant increase in resistance. A recent study has shown that both MLOC_64204 and MLOC_6065 have UDP-arabinose mutase (UAM) activity and are able to convert UDP-l-arabinopyranose to UDP-l-arabinofuranose (Hsieh et al., 2016). UDP-l-Arabinofuranose is the required form of substrate arabinose for arabinosyltransferases that attach arabinosyl residues to the xylan backbone. Given that these two genes encode enzymes that are presumed to perform the same enzymatic function, it is not unexpected that silencing single members of the family had no effect. Information on silencing mutase genes and the impact on disease resistance is lacking. However, a recent study shows that in *Brachypodium*, silencing of GT75 genes increases xylanase mediated digestibility of the cell wall by two-fold and is related to a significant decrease in cell wall ferulic acid and *p*-coumaric acid concentrations (Rancour et al., 2015).

As shown in a phylogeny of the GT8 family, the next two candidates, MLOC_19204 and MLOC_65693, are present in a cluster that contains *Arabidopsis* GT8 proteins (Figure S5) that have been shown to possess glucuronosyltransferase activity (Rennie et al., 2012) suggesting that they may be involved in adding glucuronyl residues to the xylan backbone (Figure 1). Members of the GT8 family have also been implicated in pectin biosynthesis (Mohnen, 2008), but levels of pectic polysaccharides in the walls of barley and other grasses are generally low (Stone and Fincher, 2004). As noted above for arabinosyl substitution, removal of glucuronyl substituents could also alter intermolecular interactions and enhance the strength of the walls and the papillae in such a way that the attack on cell walls by pathogens is inhibited (Mortimer et al., 2010). Although, a direct relationship between glucuronyl residues on the xylan backbone and disease resistance has not been demonstrated, the absence of glucuronyl residues in the xylan backbone achieved through double mutation of the GUX genes (GT8 members) results in weaker stem and easier cell wall digestibility with cell wall degrading enzymes, compared to the wild type (Mortimer et al., 2010). This result suggests that glucuronyl residues on heteroxylans may be required for improving the overall strength of the wall against pathogen attack, by limiting enzymatic digestibility by the pathogen.

Another candidate gene that led to an increase in penetration resistance was MLOC_64310, which belongs to the GT61 family. This gene belongs to a monocotyledon-specific phylogenetic clade that consists largely of uncharacterised genes and forms a sister clade to the Arabidopsis genes AT3G18170 and AT3G18180 (Figure S3). The importance of GT61 family in penetration resistance is strengthened further by enhanced RSI upon TIGS of two additional GT61-family members. The fact that no reciprocal infection phenotypes were observed by TIGS or over-expression of individual GT61 genes again suggests the existence of functional redundancy within the family. Several members of the GT61 gene family are likely to encode arabinofuranosyl transferases, which add arabinosyl residues to the (1,4)-β-xylan backbone (Anders et al., 2012). In grass species, the arabinosyl residues can be covalently linked to ferulic acid residues, which may undergo oxidative dimerization reactions and become covalently attached to adjacent feruloylated heteroxylan chains (Burr and Fry, 2009; Marcia, 2009). Modified susceptibility levels achieved by altering gene expression levels may be attributable to differential cross-linking of heteroxylan with other polysaccharides and phenolic compounds, since previous studies showed that in grasses, arabinosyl-ferulic acid crosslinking also improved resistance against pathogen fungi by limiting enzymatic digestibility of the modified wall (Bily et al., 2003; Santiago and Malvar, 2010; Lionetti et al., 2015).

The central goal of the current study was to identify candidate genes implicated in heteroxylan biosynthesis during powdery mildew infection that might have significant impacts on penetration resistance. The main challenge for characterizing the role of individual genes involved in heteroxylan biosynthesis in papillae is embodied in the presence of multiple genes with overlapping function within each GT family and the associated interpretative constraints. Other members of a particular family might potentially compensate in a biosynthetic process when the candidate gene is silenced. Thus, the effects of over-expression of a single gene might be masked by altered expression patterns of related genes or of other genes that might need to be co-expressed to form a functional multi-enzyme complex.

Houston et al. (2016) identified that the GT8, GT61, and GT75 families are upregulated on average across abiotic and biotic stresses in Arabidopsis and barley. Our results suggest that at least one member of each of the gene families associated with arabinoxylan biosynthesis play vital roles in plant-pathogen resistance mechanisms. This is presumably related to modified heteroxylan structures and/or altered levels of heteroxylan accumulation in papillae, both of which might provide greater mechanical strength and resistance against the physical and enzymatic mechanisms of fungal penetration. The cell wall composition and integrity could also play a role in the activation of the defense response through DAMP-signaling and pattern-triggered immunity (Malinovsky et al., 2014). If heteroxylan fragments were able to activate these pathways it would be possible to conceive that small changes in polysaccharide structure could result in the oligosaccharides released during pathogenesis not being detected by cell surface receptors.

Due to technical difficulties in labeling arabinoxylan with a range of specific antibodies in the transiently transformed cells, a direct correlation of gene expression levels and arabinoxylan biosynthesis in papillae was not possible. In order to confirm this link, further experiments will be focused on demonstrating that there is a direct link between the candidate genes described here and the biosynthesis of heteroxylan. Stable transgenic lines expressing the selected genes of interest will allow immunohistological characterization of papillae and cell wall heteroxylan, however, this will be limited to the antibodies available for different heteroxylan structures and may not provide the level of detail required to completely elucidate their role in penetration resistance. Expression and purification of the candidate genes in heterologous systems could allow *in vitro* biochemical analyses to be performed, again to demonstrate the synthase activity of each candidate gene. Nevertheless, data generated in the present study is consistent with a role for heteroxylan biosynthesis genes in plant pathogenesis and suggests that manipulation of the amounts and fine structure of heteroxylan has considerable potential for the development of disease resistant lines in the future.

## Author contributions

GF, RB, PS, and AL planned and designed the research. JC, JS, NS, SL, JR, DD, and AL performed the experiments and analyzed the data. JC, PS, GF, RB, and AL wrote the manuscript.

## Funding

The work was supported by grants from the Australian Research Council (to GF and RB) and from the German Ministry of Education and Research (BARLEY-fortress, to PS).

### Conflict of interest statement

The authors declare that the research was conducted in the absence of any commercial or financial relationships that could be construed as a potential conflict of interest.
