# Supplementary material for: Altered Expression of Genes Implicated in Xylan Biosynthesis Affects Penetration Resistance against Powdery Mildew
Source: Front Plant Sci. 2017 Mar 31;8:445. doi: 10.3389/fpls.2017.00445 (PMC5374208; doi:10.3389/fpls.2017.00445)
Supplement: Supplementary file 1 [file Table1.PDF]

Table S1. List of target genes with their corresponding microarray probeset ID.

GT family	MLOC_ID	Barley1 probeset ID	HarvEST U35 contig Nr.
2.2.1 UAM GT75	MLOC_64204	CUST_23369_PI390587928	47605
2.2.1 UAM GT75	MLOC_6065	CUST_16492_PI390587928	272
2.2.1 UAM GT75	MLOC_77094	CUST_11799_PI390587928	45357
2.2.2 Exostosin GT47	MLOC_5743	CUST_36801_PI390587928	25527
2.2.2 Exostosin GT47	MLOC_15348	CUST_36294_PI390587928	6477
2.2.2 Exostosin GT47	MLOC_14407	CUST_41062_PI390587928	22764
2.2.2 Exostosin GT47	MLOC_6311	CUST_21033_PI390587928	27431
2.2.2 Exostosin GT47	MLOC_51134	CUST_40642_PI390587928	3730
2.2.2 Exostosin GT47	MLOC_46242	CUST_27179_PI390587928	33273
2.2.2 Exostosin GT47	MLOC_61024	CUST_31113_PI390587928	8450
2.2.2 Exostosin GT47	MLOC_6274	CUST_3657_PI390587928	281
2.2.2 Exostosin GT47	MLOC_2724	CUST_33465_PI390587928	46930
2.2.2 Exostosin GT47	MLOC_64806	CUST_38117_PI390587928	16642
2.2.2 Exostosin GT47	MLOC_24334	CUST_41701_PI390587928	25168
2.2.2 Exostosin GT47	MLOC_67056	CUST_4781_PI390587928	42498
2.2.2 Exostosin GT47	MLOC_81178	CUST_20990_PI390587928	27446
2.2.2 Exostosin GT47	MLOC_50357	CUST_30211_PI390587928	23051
2.2.2 Exostosin GT47	MLOC_61178	CUST_23247_PI390587928	22628
2.2.2 Exostosin GT47	MLOC_53593	CUST_27161_PI390587928	45266
2.2.2 Exostosin GT47	MLOC_68036	CUST_18718_PI390587928	13607
2.2.2 Exostosin GT47	MLOC_59543	CUST_40775_PI390587928	48322
2.2.2 Exostosin GT47	MLOC_35	CUST_13920_PI390587928	22322
2.2.2 Exostosin GT47	MLOC_76930	CUST_17628_PI390587928	44510
2.4.1 GT8	MLOC_65693	CUST_5992_PI390587928	18727
2.4.1 GT8	MLOC_19204	CUST_23401_PI390587928	11661
2.4.1 GT8	MLOC_68075	CUST_16622_PI390587928	5357
2.4.1 GT8	MLOC_10600	CUST_3647_PI390587928	28102
2.4.1 GT8	MLOC_31668	CUST_15109_PI390587928	20445
2.4.1 GT8	MLOC_11661	CUST_32472_PI390587928	4866
2.4.1 GT8	MLOC_56389	CUST_31039_PI390587928	3448
2.4.1 GT8	MLOC_12909	CUST_38120_PI390587928	30934
2.4.1 GT8	MLOC_15026	CUST_6322_PI390587928	21405
2.4.1 GT8	MLOC_4908	CUST_40744_PI390587928	48332
2.4.1 GT8	MLOC_4421	CUST_39840_PI390587928	12915
2.4.1 GT8	MLOC_73012	CUST_35054_PI390587928	24732
2.4.1 GT8	MLOC_65292	CUST_5361_PI390587928	44181
2.4.1 GT8	MLOC_62261	CUST_12718_PI390587928	48404
2.4.1 GT8	MLOC_65730	CUST_8053_PI390587928	15254
2.4.1 GT8	MLOC_51287	CUST_20585_PI390587928	8370
2.4.1 GT8	MLOC_17998	CUST_39016_PI390587928	20126
2.4.1 GT8	MLOC_7501	CUST_11633_PI390587928	17402
2.4.1 GT8	MLOC_56677	CUST_27411_PI390587928	6778
2.4.1 GT8	MLOC_57229	CUST_37507_PI390587928	38885
2.4.1 GT8	MLOC_71562	CUST_3403_PI390587928	11863

2.4.1 GT8	MLOC_5185	CUST_14393_PI390587928	3391
2.4.1 GT8	MLOC_16176	CUST_9439_PI390587928	1095
2.4.1 GT8	MLOC_13626	CUST_11105_PI390587928	9468
2.4.1 GT8	MLOC_52341	CUST_17266_PI390587928	31512
2.4.1 GT8	MLOC_19633	CUST_39402_PI390587928	7444
2.4.2 GT61	MLOC_67646	CUST_34034_PI390587928	20792
2.4.2 GT61	MLOC_64310	CUST_21582_PI390587928	15717
2.4.2 GT61	MLOC_70966	CUST_15051_PI390587928	18749
2.4.2 GT61	MLOC_6356	CUST_37306_PI390587928	11127
2.4.2 GT61	MLOC_80855	CUST_4301_PI390587928	4292
2.4.2 GT61	MLOC_35025	CUST_37325_PI390587928	11106
2.4.2 GT61	MLOC_45236	CUST_36593_PI390587928	17373
2.4.2 GT61	MLOC_75089	CUST_4908_PI390587928	21356
2.4.2 GT61	MLOC_68728	CUST_40512_PI390587928	17353
2.4.2 GT61	MLOC_59762	CUST_36430_PI390587928	5827
2.4.2 GT61	MLOC_7745	CUST_40612_PI390587928	3760
2.4.2 GT61	MLOC_56777	CUST_36675_PI390587928	7580
2.4.2 GT61	MLOC_43588	CUST_36379_PI390587928	1849
2.4.2 GT61	MLOC_77077	CUST_22585_PI390587928	9126
2.4.2 GT61	MLOC_36764	CUST_33276_PI390587928	7900
2.4.2 GT61	MLOC_13713	CUST_21580_PI390587928	15719
2.4.2 GT61	MLOC_12296	CUST_34831_PI390587928	48932
2.4.2 GT61	MLOC_68383	CUST_9211_PI390587928	6957
2.4.2 GT61	MLOC_68968	CUST_36380_PI390587928	1848
2.4.2 GT61	MLOC_70646	CUST_23219_PI390587928	10703
2.4.2 GT61	MLOC_29067	CUST_12850_PI390587928	21808
2.4.3 GT43	MLOC_56928	CUST_1756_PI390587928	6236
2.4.3 GT43	MLOC_54026	CUST_23077_PI390587928	3935
2.4.3 GT43	MLOC_10770	CUST_38421_PI390587928	3199
2.4.3 GT43	MLOC_4722	CUST_38895_PI390587928	12379
5.2 GalT GT31	MLOC_39786	CUST_41953_PI390587928	6737
5.2 GalT GT31	MLOC_79335	CUST_33267_PI390587928	7907
5.2 GalT GT31	MLOC_16945	CUST_38747_PI390587928	13441
5.2 GalT GT31	MLOC_57943	CUST_5415_PI390587928	43392
5.2 GalT GT31	MLOC_63534	CUST_22341_PI390587928	10389
5.2 GalT GT31	MLOC_57135	CUST_37589_PI390587928	2183
5.2 GalT GT31	MLOC_48425	CUST_31987_PI390587928	28561
5.2 GalT GT31	MLOC_58820	CUST_1214_PI390587928	42941
5.2 GalT GT31	MLOC_542	CUST_37118_PI390587928	571
5.2 GalT GT31	MLOC_13798	CUST_24035_PI390587928	39385
5.2 GalT GT31	MLOC_80315	CUST_20763_PI390587928	23331
5.2 GalT GT31	MLOC_12515	CUST_19477_PI390587928	10174
5.2 GalT GT31	MLOC_34889	CUST_16218_PI390587928	27557
5.2 GalT GT31	MLOC_58820	CUST_1906_PI390587928	5738
5.2 GalT GT31	MLOC_66964	CUST_28585_PI390587928	5147
