# Supplementary material for: Altered Expression of Genes Implicated in Xylan Biosynthesis Affects Penetration Resistance against Powdery Mildew
Source: Front Plant Sci. 2017 Mar 31;8:445. doi: 10.3389/fpls.2017.00445 (PMC5374208; doi:10.3389/fpls.2017.00445)
Supplement: Supplementary file 2 [file Table2.PDF]

Table S2. Primers used to amplify the candidate genes for making the dsRNAi constructs used in Transiently Induced Gene Silencing experiments.

**Sequence Name**	**Forward Primer**	**Reverse Primer**
MLOC_5743	TAGCTCGAAAGGTGCCCTCA	TCTTGCTCCGAGAATGGAAA
MLOC_79335	GATGTGAGCATGGGCATGTG	GCCGGTATACAAGTAAGATCAGCAA
MLOC_19204	TTGGCTTGTAAAACCCGTGG	CGTGAAGGCAAAGGCAAGAG
MLOC_6065	CAAAGGCCTCATGCATCTCC	TGAGCTGACGGTCAAAAGCA
MLOC_39786	CATCCTGACGATGCCGAAG	TCCCAGGACATGACGTACCC
MLOC_65693	GCTACAAAGCGGATTGCTGG	TGGCTGCTTATACGATCCCG
MLOC_80855	TGACCAACATGGTGTTCCTCC	GTCAAGCAAAACGAGGCAGG
MLOC_6357	TATCCCCCGCTGATTACACG	GTCTCGTAGCACACCGGCTT
MLOC_67646	ATCGATCGAGCACCCTACCA	GCTACGAGAAGGCGACGAAC
MLOC_64310	ACAGCACCAACAGACGAGCA	GCAGCTCCACTACATCGGCT
MLOC_35025	CCGCATCCAACTCGCTCTT	CGGGTGTCGTTGGAGAAGTC
MLOC_70966	CGAGTGACCTGAGGCTGGAG	TCCTCTATTCCTCGGCCGTC
MLOC_64204	CAAGGAGAAGCTCGGCAAGA	CGGAAACATTAGCAGCCACA
MLOC_16945	ACCAACTGAGCCTGTCGAGC	AAGCCTCTTCTGGCCATTCC
MLOC_15348	TGTTTTCTCCAACGACGGCT	CATCTTCGTCTTTGGCAGGG
MLOC_14407	GCTCCAATCAAGAGCGTCGT	ACGCAGATGTTTCGGAAGGA
MLOC_54026	GGCTGCAAGAGAGTTGCGTT	GGCCATTCCACCACTAACCA
