# Supplementary material for: Altered Expression of Genes Implicated in Xylan Biosynthesis Affects Penetration Resistance against Powdery Mildew
Source: Front Plant Sci. 2017 Mar 31;8:445. doi: 10.3389/fpls.2017.00445 (PMC5374208; doi:10.3389/fpls.2017.00445)
Supplement: Supplementary file 3 [file Table3.PDF]

Table S3. Primers used to amplify the full coding region of the candidate genes used in transient over-expression experiments.

Sequence Name	Forward Primer	Reversed Primer
MLOC_5743	CCTCCACCCGCATTGCTA	CAGCTGGGATAGTTTGTTGGTC
MLOC_19204	ATCACAATAGCACCGGACAAG	CCTCGTGTAAAAATGTACAACAACA
MLOC_6065	CAGGGCTCGAGATCGAGAT	TCCAGGCCGTAACCATACACT
MLOC_39786	GCTTCCATTCCATCAAATCAAC	AGAACTAAAACAAGCGATCGATC
MLOC_65693	TAGCAGCGGCTTGTTGACC	CTGAATCAGCTGTCCATATGATGA
MLOC_80855	CAAGCAAGCAAGGGAGCTG	ATCTACCGAATCAGGGACGG
MLOC_6357	TGTGGAGGTCCTGTGCG	AAGCTCCCGCGATGATG
MLOC_67646	CGTCCATCCCGTTTCCATT	CCAACCAACCAAGCAGAGCTA
MLOC_64310	TAGCCGCACCCAGAGAGAG	CATGATCAGATAGGCCCGG
MLOC_35025	GCATCCCACTCCATCTGTCC	ACTGGGTATGGAGCATGAACAC
MLOC_70966	AGCAAGGCAGCAACGCAT	CAGTACGCAGAGGTTACGGTC
MLOC_64204	TCTGGGATCAGGGAGGAGAG	CACCGCCTGCTCAGTATGC
MLOC_16945	GAAACGGCTACTTCTCCTCGC	TCCTCTCGGACCAGACACAT
MLOC_15348	CTGCGGCTGCTGCAATG	GAGCAGCCAGTTGTGCG
MLOC_14407	AGCCCATTTCCAGTGGTTCT	CTCAAGGTGGAAGACGACTAGCT
MLOC_54026	CGACGAGAGAGCTTGAGAGAGT	CTGTGCTTCTTGTTGGGCC
HvSNAP34	GATCGATCTCGCCTCCGC	TAACAGCCCACGAAAGCAAATG
