# Supplementary figures and images for: Altered Expression of Genes Implicated in Xylan Biosynthesis Affects Penetration Resistance against Powdery Mildew
Source: Front Plant Sci. 2017 Mar 31;8:445. doi: 10.3389/fpls.2017.00445 (PMC5374208; doi:10.3389/fpls.2017.00445)

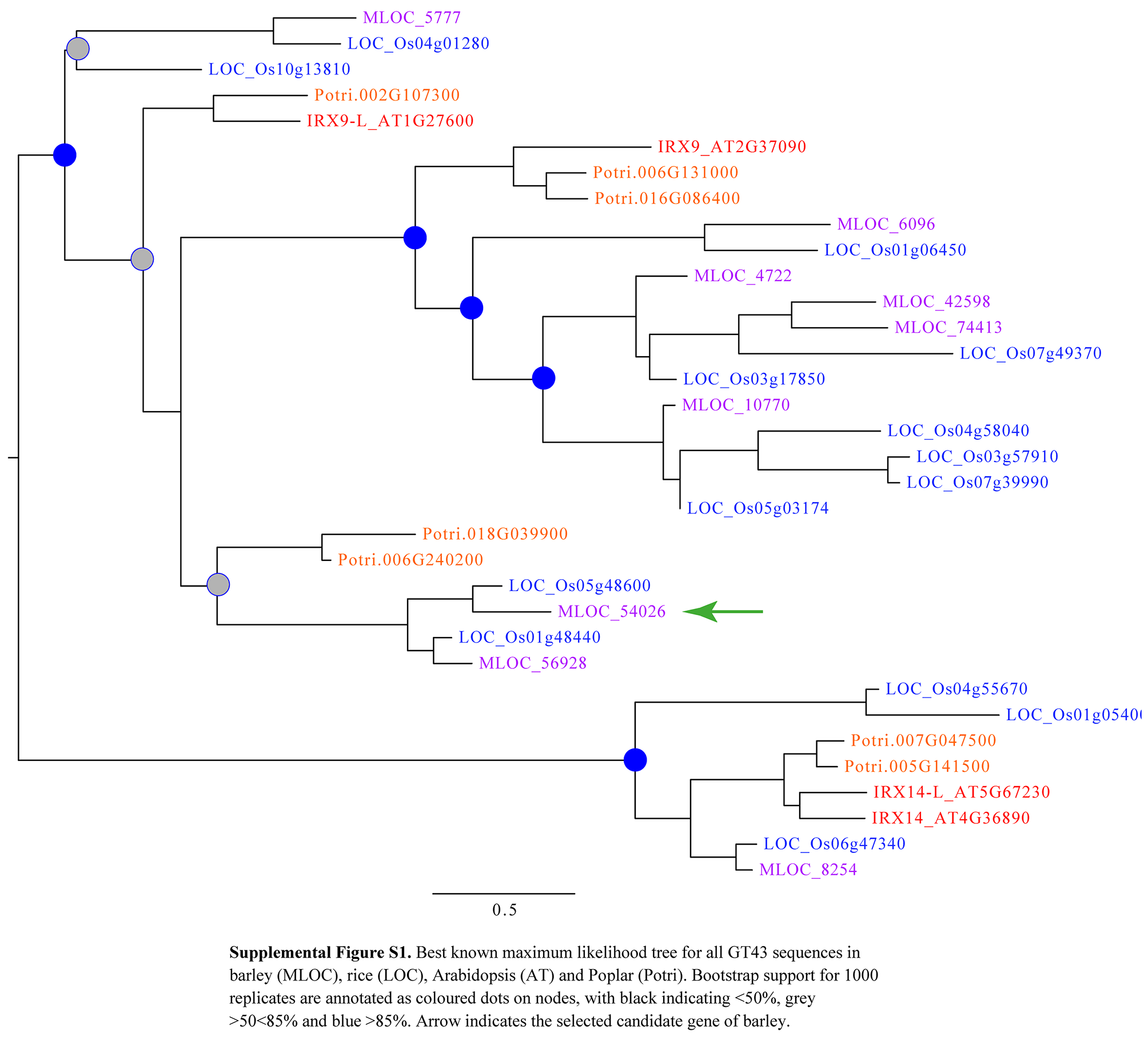

Supplement: Supplementary file 4 [file Image1.TIF]

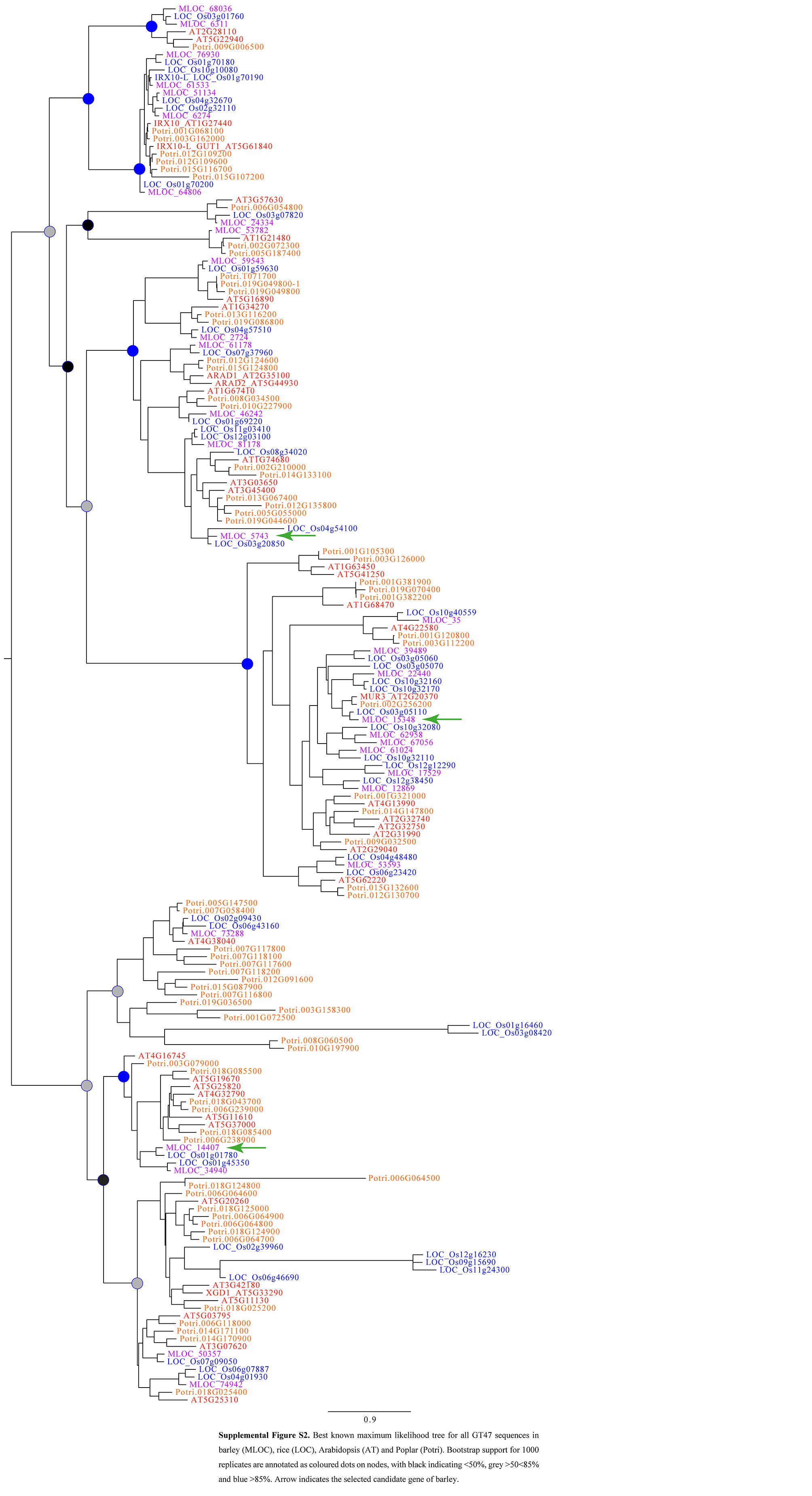

Supplement: Supplementary file 5 [file Image2.TIF]

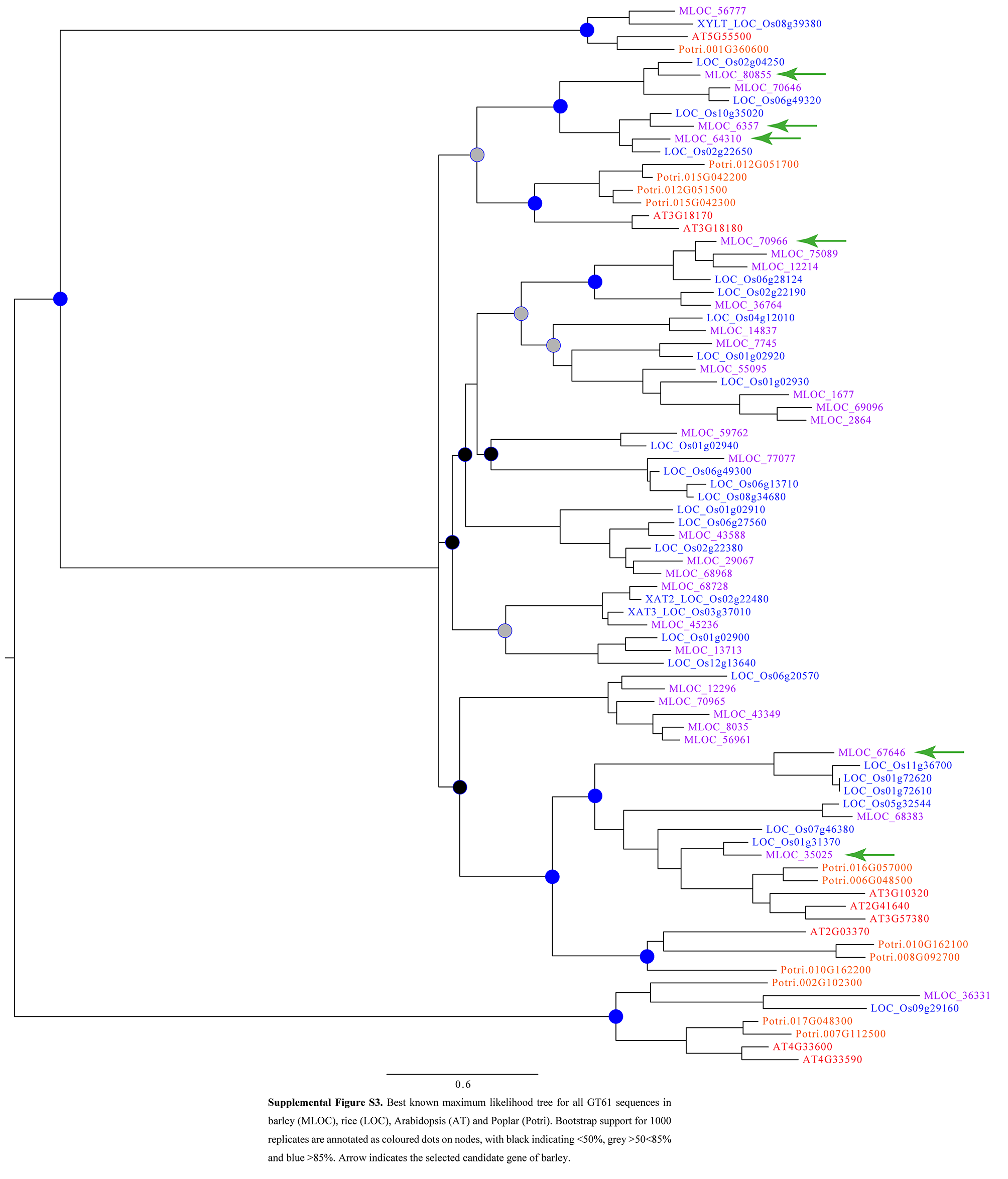

Supplement: Supplementary file 6 [file Image3.TIF]

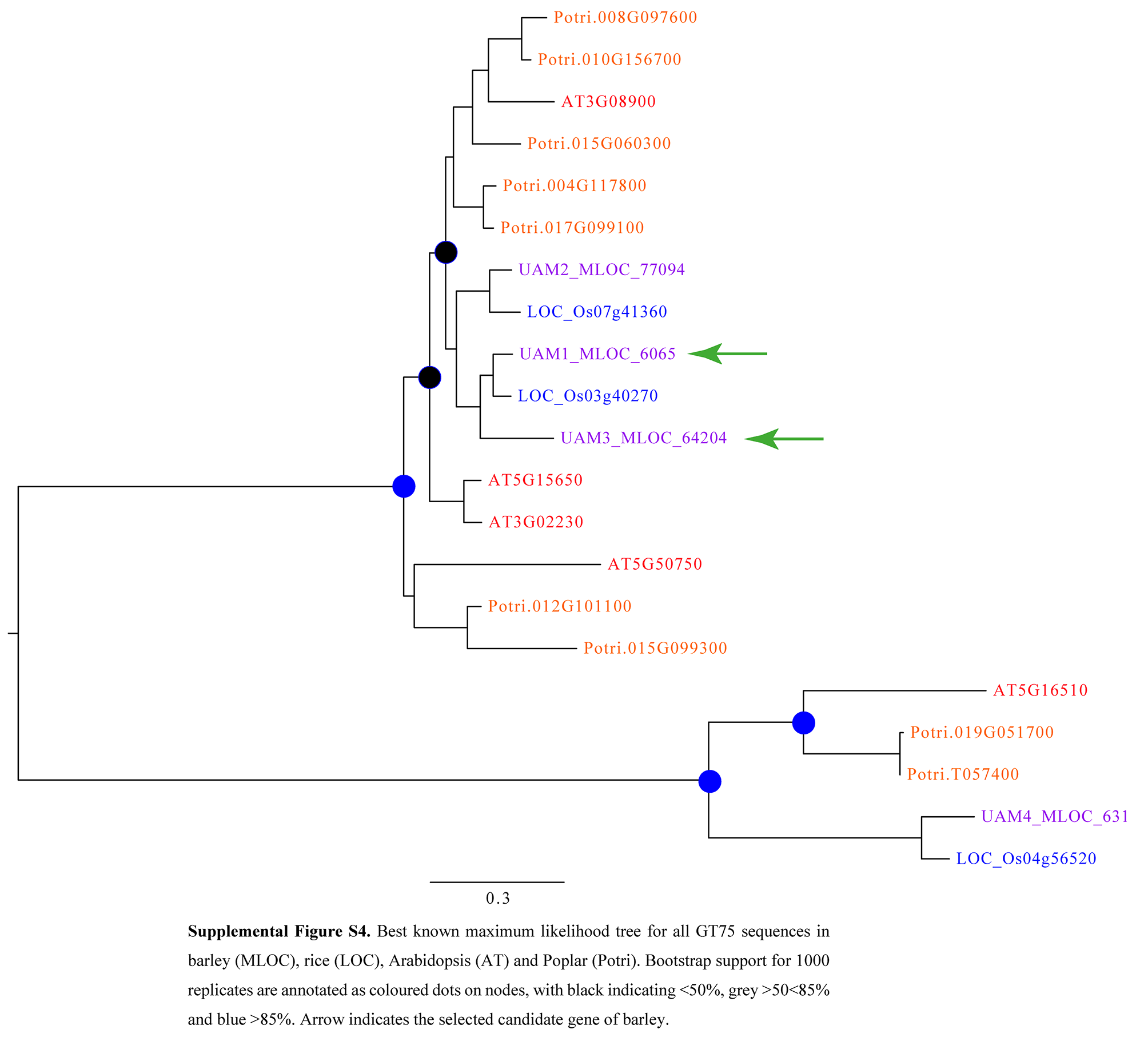

Supplement: Supplementary file 7 [file Image4.TIF]

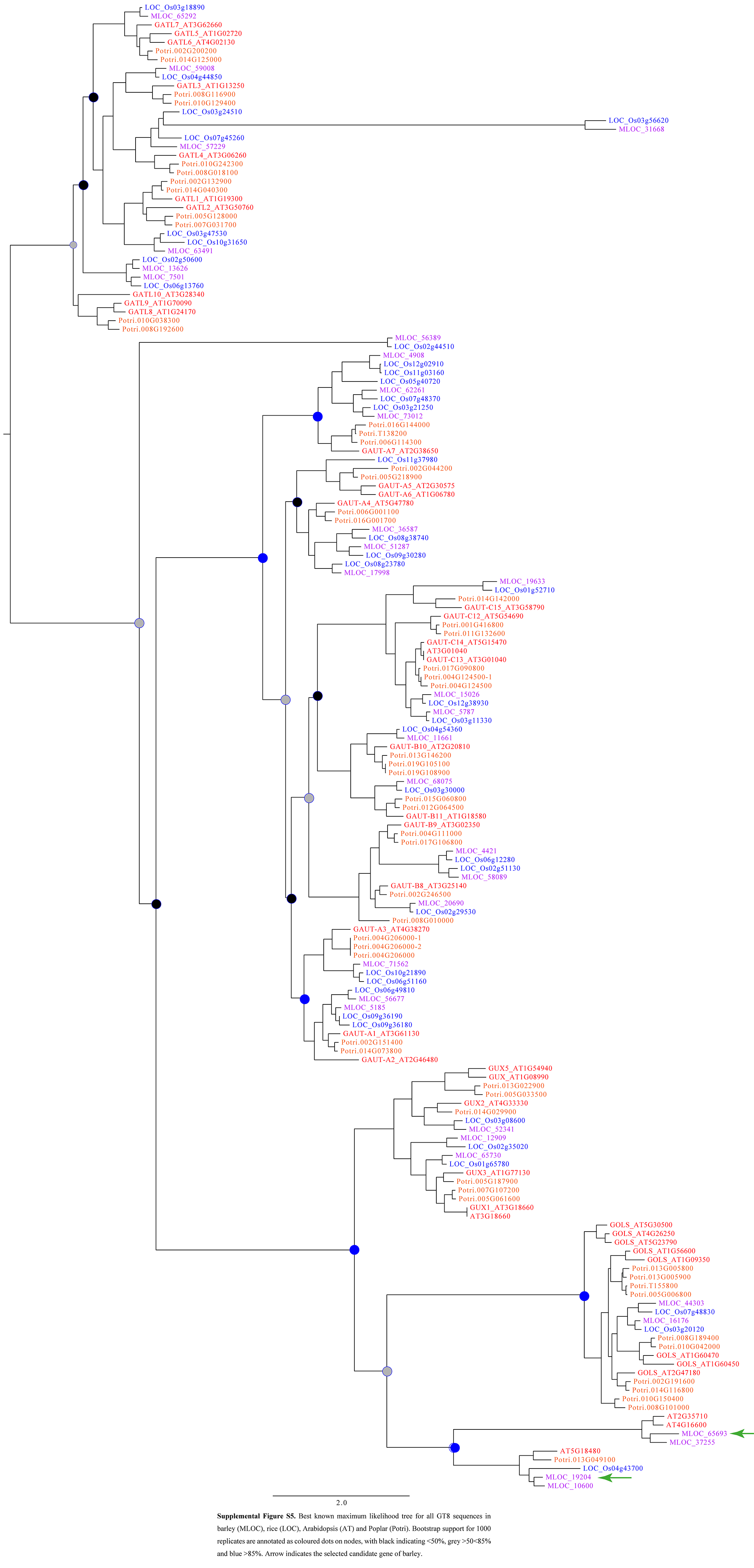

Supplement: Supplementary file 8 [file Image5.TIF]

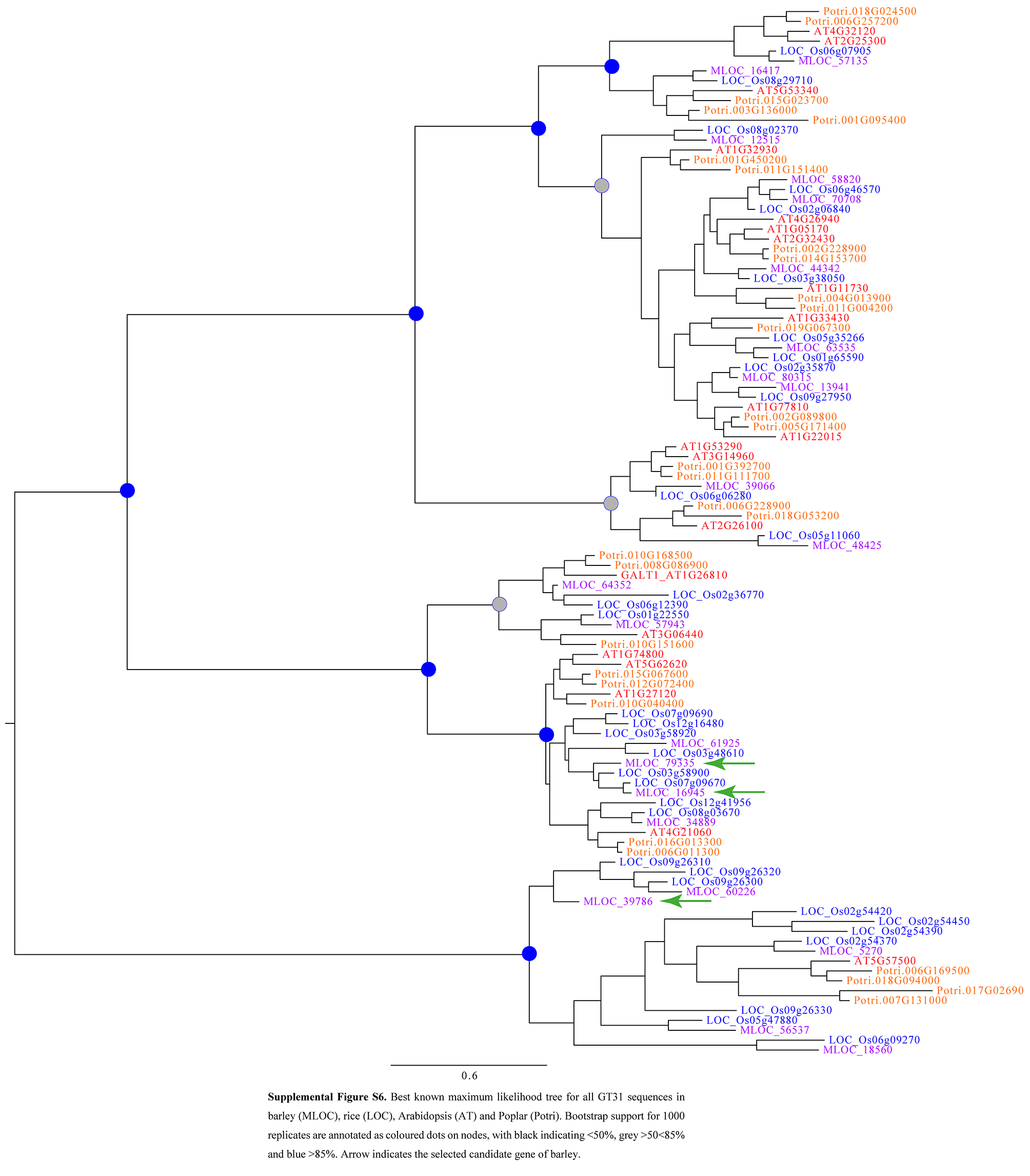

Supplement: Supplementary file 9 [file Image6.TIF]
